# Hierarchical CuO Nanorods via Cyclic Voltammetry Treatment: Freestanding Electrodes for Selective CO_2_-to-Formate Conversion

**DOI:** 10.3390/nano15171349

**Published:** 2025-09-02

**Authors:** Lili Wang, Xianlong Lu, Bangwei Deng

**Affiliations:** 1 Yangtze Delta Region Institute (Huzhou), University of Electronic Science and Technology of China, Huzhou 313001, China; 2 CMA Key Open Laboratory of Transforming Climate Resources to Economy, Chongqing 401147, China

**Keywords:** CO_2_ reduction, CuO nanorod, cyclic voltammetry treatment, freestanding electrode, formate

## Abstract

Electrochemical CO_2_ reduction reaction (CO_2_RR) represents a promising pathway for carbon neutralization. Here, we report hierarchical CuO nanorod arrays synthesized via cyclic voltammetry (CV) treatment as freestanding electrodes for selective CO_2_RR. The CV activation process generates ultrathin nanosheets on CuO nanorods, creating abundant interfaces that facilitate formate production. Optimized CV-2000-CuO achieves 42% Faradaic efficiency (FE) for formate at −1.4 V vs. RHE while suppressing hydrogen evolution reaction (HER). Comprehensive characterization reveals that CV treatment promotes partial surface reduction to metallic Cu and generates high-density grain boundaries during CO_2_RR operation. These structural features enhance CO_2_RR activity and stability compared to pristine CuO (P-CuO). This work demonstrates a novel electrode engineering strategy combining freestanding architecture with electrochemical activation for efficient CO_2_-to-formate conversion.

## 1. Introduction

Electrochemical CO_2_ reduction reaction (CO_2_RR) represents a promising pathway for converting greenhouse gases into value-added chemicals and fuels, thereby addressing both environmental sustainability and energy storage challenges [1,2,3,4,5,6,7,8]. Copper (Cu)-based catalysts are uniquely capable of producing multi-carbon (C_2+_) products such as ethylene and ethanol, yet their practical deployment is hindered by challenges in selectivity control, high overpotentials, and structural instability under operating conditions [3,9,10,11,12,13,14,15,16,17]. The dynamic reconstruction of Cu surfaces during CO_2_RR further complicates the identification of active sites [10,18,19,20].

Recent advances in copper catalysts emphasize morphological and compositional engineering to enhance C_2+_ yields [21,22,23,24,25,26,27]. For instance, crystalline/amorphous dual-phase Cu structures promote *CO coverage via chloride adsorption in neutral media, achieving 81% Faradaic efficiency (FE) for C_2+_ products [28]. Similarly, carbonate-shell-regulated CuO reconstruction enriches grain boundaries, boosting ethylene selectivity to 82.8% [29], while molecular modifiers like tannic acid optimize Cu^δ+^/Cu^0^ interfaces for C_2_H_4_ formation [30]. Among these factors, grain boundaries play a crucial role in enhancing catalytic performance. A grain boundary is the interface between adjacent grains in a polycrystalline material and can be classified into low-angle grain boundaries (<15°) and high-angle grain boundaries (>15°) according to the crystallographic orientation difference. Owing to atomic undercoordination and lattice strain, grain boundary regions possess high surface energy, which facilitates reactant adsorption and intermediate activation. The unsaturated coordination atoms and strain fields at grain boundaries can synergistically modulate the adsorption energy of reaction intermediates, thereby significantly improving the selectivity and yield of C_2_^+^ products [31]. Additionally, Xia et al. systematically reviewed the latest advances, reaction mechanisms, and challenges of copper-based alloy catalysts—including conventional alloys, high-entropy alloys, and single-atom alloys in the CO_2_RR. By doping with metals such as Ag, Au, and Pd, they effectively modulated the electronic structure of copper and the adsorption energies of reaction intermediates, successfully breaking the traditional linear scaling relationships and significantly enhancing catalytic selectivity and activity [32]. Despite these successes, formate production on Cu-based catalysts is often marginalized, with only sparse reports of Cu-based systems exceeding 50% FE [33,34,35,36,37,38,39,40,41,42,43,44]—typically requiring Sn or S heteroatoms [45,46] or molecular additives like cetyltrimethylammonium bromide (CTAB) [43]. For instance, Li et al. [45] reported a novel Cu_2_O/CuS catalyst that could both enhance the activity and stability of formate production, which outperforms most Cu, Cu_2_O or CuS catalysts. The sulfide phase proved to have an important role in tuning the selectivity of CO_2_ reduction. On the other hand, given previous density functional theory calculations have shown that the key intermediate (HCOO*) for formate evolution usually binds strongly to the Cu catalyst surface and the desorption of HCOO* has been proved the rate-limiting step of CO_2_RR to formate. The CTAB modification of Cu surface therefore has also been demonstrated to facilitate the desorption of HCOO* and greatly improved formate formation [43]. It should be noted that Cu-based catalysts without any heteroatoms or modifiers usually show much lower selectivity and activity of formate formation. For example, the leaf-like CuO nanosheets with only 20% FE of formate evolution [42], CuO-derived hierarchical nanostructures with a maximum formate FE of 38%, and the Cu(OH)_2_-derived nanowires exhibited only 23% FE [35], etc. Therefore, there is urgent need to investigate the true activity site of formate formation on Cu-based catalysts and help to design novel structures for CO_2_-to-formate. Moreover, conventional powder-based catalysts suffer from binder-induced impedance and inhomogeneous active site distribution, limiting reproducibility and mass transport. Freestanding electrodes, though promising [47,48,49,50,51], have not yet leveraged in situ cyclic voltammetry (CV) treatment for surface engineering. This gap underscores the need for integrated approaches that combine tunable reconstruction methods with freestanding architectures to unlock formate-selective pathways.

In this work, we reported CV-activated CuO (CV-CuO) nanorod arrays as a novel freestanding catalyst for efficient CO_2_ conversion. The CV process generates hierarchical CuO nanosheets on pristine CuO (P-CuO) nanorods, creating abundant interfaces that favor CO_2_RR, with the CV-2000-CuO sample shows the highest FE of formate formation (42%) and most suppressed hydrogen evolution reaction (HER). The X-ray diffraction (XRD) results showed that both the high valence state of Cu in the bulk phase of both P-CuO and CV-CuO are nearly reduced to metallic Cu during activated process. However, the ex situ X-ray photoelectron spectroscopy (XPS) results suggested that both of the two samples are partially oxidized on the surface, while the CV-CuO sample exhibited more metallic Cu compared to P-CuO. The transmission electron microscopy (TEM) and Scanning electron microscopy (SEM) with an electron backscatter diffraction (EBSD) model further demonstrated the CV-CuO generated more grain boundaries and interface during CO_2_RR process, which might be the true active site for the improved performance of CO_2_RR. This work not only demonstrates a freestanding electrode strategy but also expands the functional scope of CV engineering into CO_2_RR catalysis.

## 2. Materials and Methods

### 2.1. Materials

Polycrystalline copper foil (poly-Cu, 25 µm thickness, ≥99.99%) was supplied by Alfa Aesar. potassium hydroxide (KOH, AR, ≥85%), ammonium persulfate ((NH_4_)_2_S_2_O_8_, AR, ≥98%), Sodium hydroxide (NaOH, AR, ≥98%), hydrochloric acid (HCl, 37 wt%), acetone (AR), and ethanol (AR) were procured from Adamas. Deionized water (DI water, 18.2 MΩ cm resistivity) was generated using a Milli-Q purification system (Nanjing Yuheng Instrument Equipment Co., Ltd., Nanjing, China). All chemicals were utilized as received without additional purification.

### 2.2. Synthesis of Hierarchical CuO Nanorod Arrays on Polycrystalline Cu Foil

The hierarchical CuO nanorod arrays were synthesized through a sequential process involving hydrothermal growth, thermal annealing, and cyclic voltammetry treatment, adapted from a reported method with a key modification in the substrate [46]. Polycrystalline copper foil (poly-Cu, thickness: ~100 µm) was employed as the substrate instead of copper foam.

Substrate Pretreatment: Rectangular pieces of poly-Cu foil (typically 2.0 × 4.0 cm^2^) underwent sequential ultrasonic cleaning in 1 M HCl, acetone, ethanol, and deionized (DI) water (5 min per solvent) to eliminate surface oxides and contaminants. The cleaned foils were then dried thoroughly in an oven at 60 °C.

In Situ Growth of Cu(OH)_2_ Nanorod Arrays: An aqueous reaction solution was prepared by mixing equal volumes of 2.5 M NaOH (15.0 mL) and 0.125 M (NH_4_)_2_S_2_O_8_ (15.0 mL) under constant stirring until homogeneity was achieved. The pretreated poly-Cu foil was submerged in this solution at ambient temperature (~25 °C) for 20 min. During this step, the copper surface is oxidized by persulfate (S_2_O_8_^2−^) in the alkaline medium, leading to the formation of Cu^2+^ ions which subsequently precipitate as wafter-blue Cu(OH)_2_ nanorods directly on the foil surface. After reaction, the foil was removed, rinsed copiously with DI water and ethanol, and dried at 60 °C in a vacuum oven.

Thermal Conversion to CuO Nanorod Arrays (P-CuO): The as-prepared in situ growth of Cu(OH)_2_ nanorod arrays samples were converted to CuO via a controlled thermal annealing process under a nitrogen atmosphere. The transformation involved a two-stage heating profile in a tube furnace: first at 120 °C for 3 h to facilitate dehydration, followed by a ramp to 180 °C at 2 °C min^−1^ and holding for an additional 3 h to promote crystallization. The initial ramp rate to 120 °C and cooling rates were maintained at 5 °C min^−1^. This process yielded CuO nanorod arrays supported on poly-Cu foil, designated as P-CuO.

Cyclic Voltammetry (CV) Treatment: The P-CuO samples were subjected to electrochemical treatment using cyclic voltammetry (CV) in a three-electrode configuration with 6 M KOH electrolyte. The P-CuO served directly as the working electrode, while a platinum foil counter electrode and a Hg/HgO reference electrode were employed. CV scans were performed within a potential window of 0 V to +0.5 V (vs. Hg/HgO) at a scan rate of 50 mV s^−1^ for specific cycle numbers (2000, 4000, or 8000 cycles). The potential range of 0 to +0.5 V (vs. Hg/HgO) was selected to induce mild redox-driven morphological reconstruction of CuO while preventing over-reduction or bulk phase collapse. This potential window enables the dynamic formation of ultrathin secondary nanosheets on the CuO nanorod surface, thereby enhancing surface area and catalytic active site exposure. The resulting samples are denoted as CV-X-CuO, where X represents the number of CV cycles applied (e.g., CV-2000-CuO, CV-4000-CuO, CV-8000-CuO). The pristine sample without CV treatment is referred to as P-CuO.

### 2.3. Characterization

Scanning electron microscopy (SEM, Hitachi SU8020, 10 kV acceleration voltage, Hitachi High-Tech Corporation, Tokyo, Japan) with an electron backscatter diffraction (EBSD) and transmission electron microscopy (TEM, JEOL JEM-2100F, 200 kV, Tokyo, Japan) coupled with selected-area electron diffraction (SAED) and energy-dispersive X-ray spectroscopy (EDS) mapping were employed to analyze nanorod morphology, crystallinity, and elemental distribution. X-ray diffraction (XRD) patterns were acquired on a Bruker D8 Advance diffractometer (Bruker Corporation, Karlsruhe, Germany) (Cu Kα radiation, λ = 1.5418 Å) over a 2θ range of 10°–80° to determine phase composition and crystal structure. X-ray photoelectron spectroscopy (XPS) was conducted on a Thermo Scientific K-Alpha+ spectrometer (Thermo Fisher Scientific Inc., Waltham, MA, USA) (monochromatic Al Kα source, 1486.6 eV). Binding energies were calibrated against the C 1s peak (284.8 eV). Peak deconvolution was performed using Avantage software (5.9931) with Shirley backgrounds.

### 2.4. Electrochemical Measurements

All CO_2_ reduction reaction (CO_2_RR) measurements were conducted in a custom two-compartment H-cell electrolyzer. The anodic and cathodic compartments were physically separated by a Nafion 117 proton exchange membrane, which was pre-treated by sequential boiling in H_2_O_2_ (3wt%), deionized water, H_2_SO_4_ (5wt%), and deionized water again (each step for 1 h) to ensure proton conductivity and minimize ion crossover. The catalyst materials under investigation, namely P-CuO and CV-X-CuO, were fabricated into electrodes with a defined geometric surface area of 1.0 cm × 1.0 cm (1.0 cm^2^). A key aspect of the electrode preparation was the direct use of these catalyst samples as the working electrodes. No polymeric binders (e.g., Nafion) or conductive additives (e.g., carbon black) were employed in the electrode fabrication. This binder-free approach eliminates potential interference from organic binders in product distribution analysis and ensures the measured activity and selectivity are solely attributable to the intrinsic properties of the P-CuO and CV-X-CuO catalysts. The electrodes were mechanically secured to ensure good electrical contact with the potentiostat lead. 0.1 M KHCO_3_ aqueous solution (pH ≈ 8.3) was saturated with high-purity CO_2_ (≥99.999%) for ≥30 min before all measurements to ensure saturation. Crucially, the CO_2_ purge was maintained continuously throughout the entire duration of the electrochemical experiments to replenish consumed CO_2_ and maintain a constant concentration near the electrode surface (The CO_2_ flow rate was set to 30 sccm), preventing mass transfer limitations from dominating the observed performance.

Prior to evaluating the steady-state CO_2_RR performance, each working electrode underwent an in situ electrochemical activation step. This activation was performed by applying a constant potential of −1.2 V vs. Ag/AgCl to the working electrode for 30 min. The activation process continued until the recorded current density reached a stable plateau, indicating a relatively steady surface state of the catalyst had been achieved. Electrochemical tests utilized a CHI 760E potentiostat. Linear sweep voltammetry (LSV) and chronoamperometry (CA) were conducted at room temperature. Potentials were measured against a leak-free Ag/AgCl reference electrode (saturated KCl solution) and converted to the reversible hydrogen electrode (RHE) scale using: *E* (vs. RHE) = *E* (vs. Ag/AgCl) + 0.210 V + 0.059 × pH. No ohmic drop was compensated. Gaseous products (H_2_, CO, CH_4_, C_2_H_4_) were quantified online during CA tests via gas chromatography (GC9790Plus, FULI INSTRUMENTS, Taizhou City, Zhejiang Province, China) equipped with thermal conductivity (TCD) and flame ionization (FID) detectors, using Ar carrier gas. Liquid products were analyzed by Nuclear Magnetic Resonance spectroscopy (NMR, AVANCE NEO 400 MHz, Bruker BioSpin GmbH, Fällanden, Switzerland). Faradaic efficiencies (FEs) were calculated as:

$${\mathrm{FE}}_{\mathrm{product}\;}(\%)\;=\;n_{\mathrm{product}}*F*n_{e}/Q_{\mathrm{total}}*100\%$$

where *n_product_* is moles of product, *F* is a constant of Faraday, *n_e_* is electrons transferred per molecule, and *Q_total_* is total charge passed.

## 3. Results and Discussion

**Figure 1:**
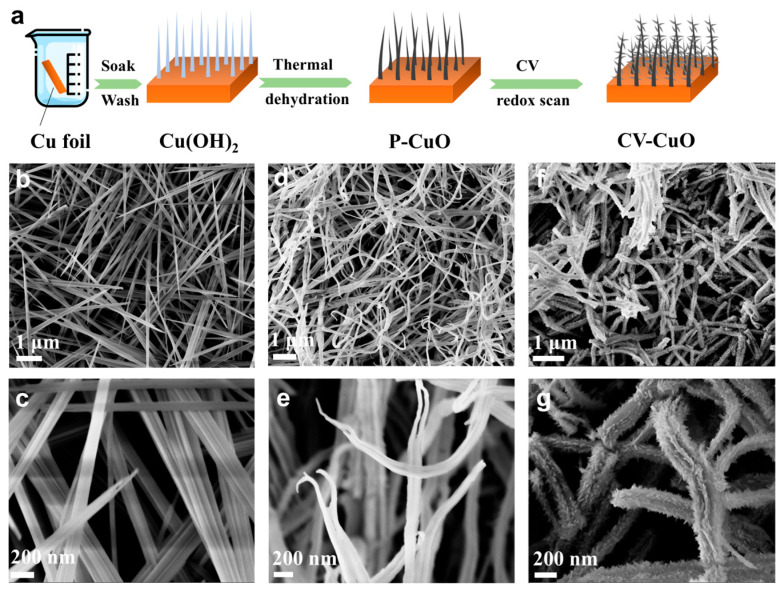
(**a**) Schematic illustration of the synthesis processes of CV-CuO nanorod arrays. SEM images of as prepared (**b**,**c**) Cu(OH)_2_, (**d**,**e**) P-CuO and (**f**,**g**) CV-CuO nanorod arrays at different magnifications.

Figure 1a schematically depicts the synthesis pathway for CV-CuO nanorod arrays. The process commences with the in situ growth of Cu(OH)_2_ nanorod arrays directly onto a Cu foil substrate at ambient temperature. This initial formation is driven by the chemical treatment reaction:

Cu + 4NaOH + (NH_4_)_2_S_2_O_8_ → Cu(OH)_2_ + 2Na_2_SO_4_ + 2NH_3_ + 2H_2_O

Under the alkaline conditions provided by NaOH, the Cu surface is oxidized by the persulfate anions (S_2_O_8_^2−^), first generating Cu^2+^ ions which rapidly precipitate as Cu(OH)_2_ nanorods. Subsequently, these Cu(OH)_2_ precursor arrays undergo a controlled thermal dehydration process (involving two distinct heating stages) to yield pristine CuO (P-CuO) nanorod arrays. The final step involves subjecting the P-CuO nanowires to cyclic voltammetry (CV) treatment. This electrochemical treatment, conducted in 6 M KOH electrolyte over a 0–0.5 V potential range, transforms the material into the target CV-CuO nanorod arrays.

The morphological evolution throughout the synthesis was characterized using SEM. Figure 1b,c and Figure S1 reveal that the as-grown Cu(OH)_2_ nanorods possess a well-defined array structure, with individual rods averaging approximately 200 nm in diameter and ~10 μm in length. Following thermal calcination to form P-CuO (Figure 1d,e), the fundamental nanorod array architecture is largely retained, although some degree of curling is observed, likely due to dehydration-induced stress. More interestingly, a significant morphological transformation occurs upon CV treatment. As exemplified by the CV-2000-CuO sample (Figure 1f,g), the CV treatment induces the formation of a distinct hierarchical structure. The surface of the original CuO nanorods appears to partially reconstruct, generating a dense and uniform coating of ultra-thin secondary nanosheets. SEM analysis of samples subjected to different numbers of CV cycles (2000, 4000, 8000; Figure S2) indicates that while the overall hierarchical morphology is consistent, the thickness of the secondary nanosheet layer increases with the cycle number. This intricate hierarchical architecture, comprising primary nanorods decorated with secondary nanosheets, is anticipated to significantly enhance the material’s specific surface area and expose a greater density of catalytically active sites compared to the P-CuO.

**Figure 2:**
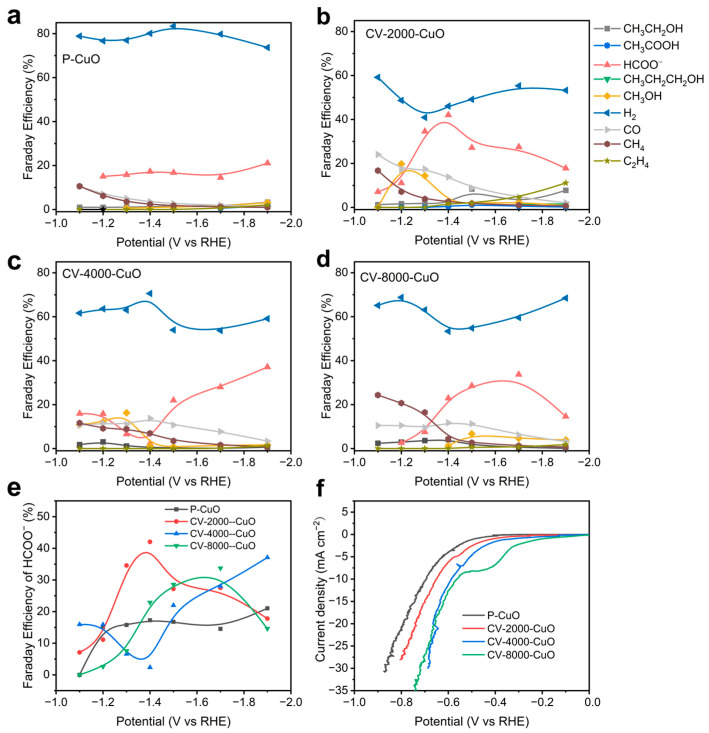
The potential-dependent FE curves of gaseous and liquid products of (**a**) P-CuO, (**b**) CV-2000-CuO, (**c**) CV-4000-CuO, and (**d**) CV-8000-CuO nanorod arrays. The corresponding (**e**) potential-dependent FE curves of HCOO^−^ and (**f**) LSV curves under CO_2_ atmosphere.

We subsequently evaluated the CO_2_RR performance of P-CuO and CV-CuO samples subjected to different numbers of CV cycles (2000, 4000, and 8000). Electrochemical tests were conducted in a three-electrode H-cell using 0.1 M KHCO_3_ electrolyte pre-saturated with CO_2_ for 30 min. All catalysts underwent electrochemical pretreatment at −1.2 V vs. Ag/AgCl for ≥30 min to achieve a stable current plateau, ensuring complete activation and maximal exposure of active sites prior to CO_2_RR measurements. Gas and liquid products were quantified by gas chromatography (GC) and nuclear magnetic resonance (NMR) spectroscopy, respectively (Figure S3). As shown in Figure 2a–f, P-CuO primarily produced H_2_ with minor formate (HCOO^−^), whereas CV-CuO exhibited marked suppression of H_2_ production alongside concomitant enhancement of formate selectivity. Figure 2e and Figure S4 compare the potential-dependent FE trends for formate and H_2_ across CV-CuO catalysts with varying cycle numbers. Notably, CV-2000-CuO achieved peak performance at −1.4 V vs. RHE, delivering the highest formate FE (42%) and lowest H_2_ FE (40%). Linear sweep voltammetry (LSV) curves in Figure 2f further confirmed superior activity for all CV-CuO catalysts, evidenced by positively shifted onset potentials for CO_2_ reduction. Moreover, the CV-2000-CuO catalyst also show much higher current density during stability tests for over 20 h, further demonstrated the effectiveness of CV activated method in promoting the activity of CO_2_RR (Figure S5).

**Figure 3:**
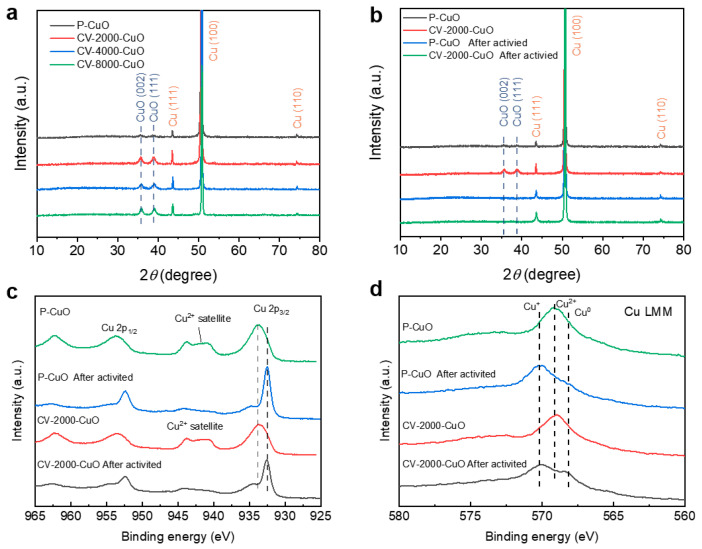
XRD and XPS analysis of catalysts before and after activation (**a**) The XRD of as prepared catalysts (P-CuO, CV-2000-CuO, CV-4000-CuO, and CV-8000-CuO). (**b**) XRD comparison of P-CuO and CV-2000-CuO before and after activation. XPS results of (**c**) Cu 2p and (**d**) Cu LMM of P-CuO and CV-2000-CuO before and after activation.

To elucidate the origin of performance enhancement in CV-CuO catalysts, we performed phase analysis via XRD on as-prepared P-CuO and CV-CuO samples (Figure 3a). All patterns exhibited characteristic CuO peaks at 35.5° (002) and 38.7° (111) alongside metallic Cu substrate signals at 43.3° (111), 50.4° (200), and 74.1° (220), confirming that CV activation preserved the crystalline structure of CuO nanorod arrays on Cu substrates. Post-activation XRD analysis of P-CuO and CV-2000-CuO (Figure 3b) revealed complete disappearance of CuO reflections with only metallic Cu peaks remaining, indicating bulk reduction of Cu^2+^ to Cu^0^ during electrochemical pretreatment. Complementary ex situ XPS analysis revealed a negative binding energy shift in the Cu 2p_3_/_2_ main peak accompanied by attenuated satellite features (Figure 3c), collectively confirming substantial depletion of surface Cu^2+^ species in both activated catalysts. Crucially, Cu LMM Auger spectra analysis uncovered distinct valence distributions (Figure 3d): significant attenuation of Cu^2+^ spectral features coupled with emerging Cu^+^ and Cu^0^ signatures confirmed substantially higher Cu^0^ content in activated CV-2000-CuO relative to P-CuO. This electronic restructuring correlates with facilitated CO_2_RR kinetics, attributable to enhanced exposure of catalytic active sites within its unique hierarchical architecture.

**Figure 4:**
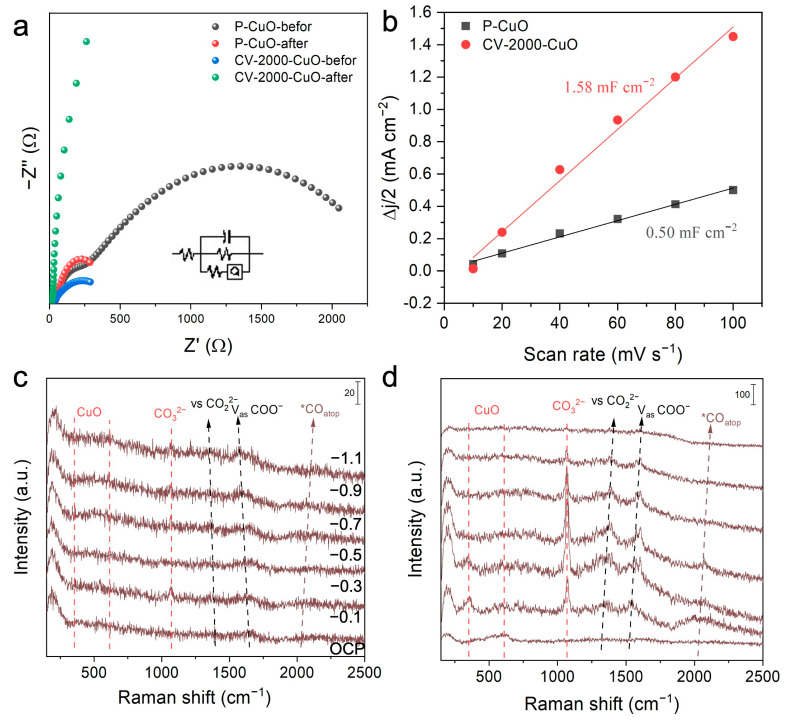
(**a**) Electrochemical impedance spectroscopy (EIS) of the samples. (**b**) Double-layer capacitance (C_dl_) measurements. (**c**,**d**) In situ Raman spectra of P-CuO and CV-2000-CuO.

BET surface area analysis revealed that both CV-2000-CuO and P-CuO catalysts lack mesopores and micropores. However, CV-2000-CuO exhibited a significantly higher specific surface area (0.0922 m^2^ g^−1^) compared to P-CuO (0.0457 m^2^ g^−1^) (Figure S6). Charge transfer resistance characterization further demonstrated the superior electrochemical performance of CV-2000-CuO. Prior to CO_2_RR testing, CV-2000-CuO already displayed lower solution resistance (R_s_) and charge transfer resistance (R_ct_) than P-CuO. Post-testing, both samples exhibited decreased resistances, with a more pronounced reduction for P-CuO; notably, CV-2000-CuO achieved a further decrease (Figure 4a). This indicates that the CV treatment significantly enhanced the conductivity and interfacial reaction kinetics of CV-2000-CuO. Consistent with this, the electrochemical active surface area (ECSA), proportional to the double-layer capacitance (C_dl_), was markedly higher for CV-2000-CuO than for P-CuO (Figure 4b and Figure S7), suggesting more exposed active sites and a surface more favorable for reactant adsorption and reaction. In situ Raman spectroscopy provided mechanistic insights: characteristic CuO peaks weakened and disappeared with increasing applied potential for both catalysts, indicating reduction to metallic Cu (Figure 4c,d). Crucially, CV-2000-CuO exhibited stronger carbonate signals than P-CuO, while neither sample showed obvious bicarbonate peaks. Concurrently, adsorption features of formate intermediates (COO^−^) and atop-bound CO (CO_atop_) were observed (Figure 4c,d), further confirming formate as the primary product in this catalytic system.

**Figure 5:**
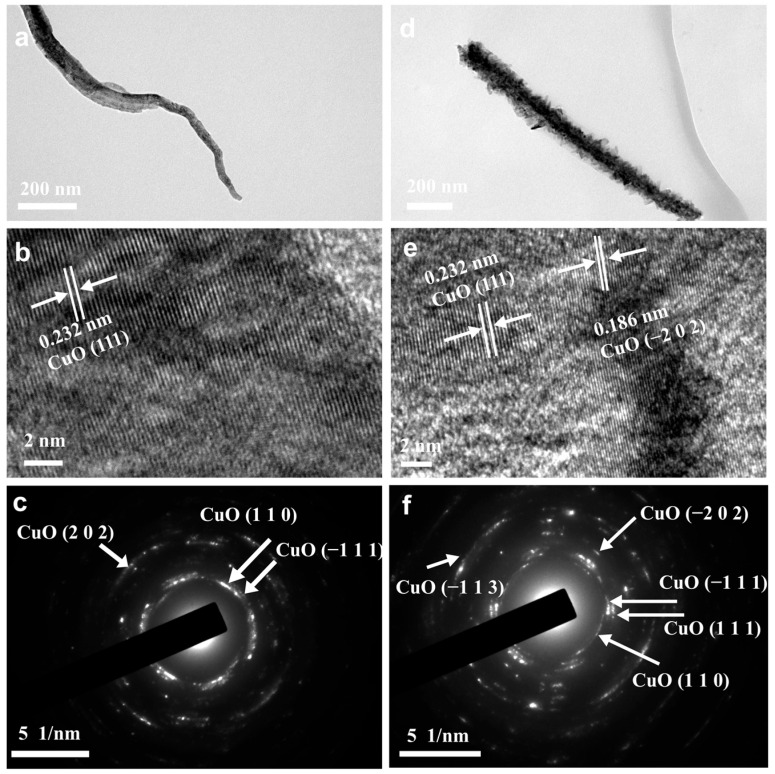
The high-resolution TEM images and corresponding SAED patterns of prepared catalysts (**a**–**c**) P-CuO and (**d**–**f**) CV-2000-CuO.

To assess the nanoscale morphological evolution induced by CV activation, high-resolution TEM (HRTEM) analysis was performed on as-prepared P-CuO and CV-2000-CuO catalysts (Figure 5 and Figure S8). Both catalysts retained nanorod frameworks consistent with SEM observations (Figure 1). P-CuO nanorods exhibited smooth surfaces (Figure 5 and Figure S8a,b), while CV-2000-CuO displayed well-defined hierarchical nanostructures epitaxially grown on nanorod backbones (Figure 5d and Figure S8c,d). HRTEM images and corresponding selected-area electron diffraction (SAED) patterns confirmed crystalline characteristics of CuO for both catalysts, exposing dominant CuO facets including (111), (110), and (−202) (Figure 5b–f). Crucially, CV-2000-CuO exhibited broader crystallographic orientation diversity, evidenced by complex lattice arrangements in HRTEM (Figure 5e) and SAED patterns (Figure 5f).

Post-electrochemical-activation SEM characterization (Figure S9a–f) revealed distinct morphological evolution pathways under cathodic polarization: P-CuO maintained nanorod arrays with increased surface roughness (Figure S9a–c), whereas CV-2000-CuO preserved hierarchical features despite partial structural densification (Figure S9d–f). After extended CO_2_RR operation, P-CuO underwent significant morphological degradation exhibiting melting-like transformation (Figure S10a–c), contrasting sharply with CV-2000-CuO’s retained hierarchical framework despite localized nanosheet coalescence (Figure S10d–f). These findings demonstrate the superior structural integrity of CV-activated architectures under electrochemical stress.

**Figure 6:**
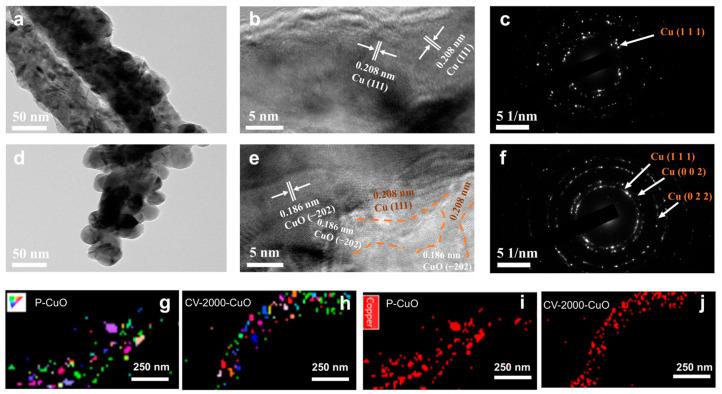
The high-resolution TEM images and corresponding SAED patterns of catalysts after activation (**a**–**c**) P-CuO and (**d**–**f**) CV-2000-CuO. The corresponding SEM-EBSD mapping of (**g**,**h**) X axis involving Cu (001), Cu (101), and Cu (111) and (**i**,**j**) Copper element distribution.

Post-electrochemical-activation microstructural characterization (Figure 6) unveiled fundamental distinctions in nanoscale architecture between CV-activated and pristine catalysts: HRTEM coupled with SAED analysis demonstrated that CV-2000-CuO developed high-density grain boundaries decorated with 10–30 nm metallic copper nanoparticles. These nanoparticles exhibited mixed crystallographic orientations, predominantly exposing (111), (002), and (022) lattice planes as evidenced by distinct diffraction spots (Figure 6d–f). This multifaceted configuration contrasts sharply with P-CuO, which formed larger crystalline domains (>50 nm) dominated by low-energy Cu (111) facets. EBSD mapping (Figure 6g–j and Figure S11) quantitatively confirmed enhanced grain boundary density and homogeneous Cu distribution in CV-2000-CuO, directly correlating with its optimized catalytic performance through increased active site accessibility.

## 4. Conclusions

We developed hierarchical CuO nanorod arrays via CV treatment as efficient freestanding electrodes for selective CO_2_-to-formate conversion. The optimized CV-2000-CuO electrode achieves 42% formate FE at −1.4 V vs. RHE, significantly outperforming pristine CuO. Structural characterization demonstrates that CV processing generates: (a) metastable Cu species with higher surface Cu^0^ content, and (b) high-density grain boundaries during CO_2_RR operation. These features collectively enhance * CO_2_ intermediate stabilization and suppress HER. The preserved hierarchical structure during long-term operation further confirms the robustness of CV-engineered electrodes. This work establishes CV treatment as a powerful tool for designing interface-rich freestanding electrodes, opening new pathways for selective CO_2_RR catalyst design.

## Data Availability

The data that support the findings of this study are available from the corresponding author upon reasonable request.

## Supplementary Materials

The following supporting information can be downloaded at https://www.mdpi.com/article/10.3390/nano15171349/s1, Figure S1: SEM images of as prepared Cu(OH)_2_ nanorod arrays at different magnifications; Figure S2: SEM images of as prepared CV-CuO nanorod arrays at different magnifications; Figure S3: Calibration curves for 1D 1H NMR quantitative analysis; Figure S4: The corresponding potential-dependent FE curves of H_2_; Figure S5: The stability test for P-CuO and CV-2000-CuO nanorod arrays electrodes; Figure S6: The high resolution TEM images of prepared catalysts; Figure S7: SEM images at different magnifications after electrochemical activated for 30min; Figure S8: SEM images at different magnifications after CO_2_RR; Figure S9: The SEM-SAED patterns of prepared catalysts.

## Abbreviations

The following abbreviations are used in this manuscript:

CO_2_RR	Electrochemical CO_2_ reduction reaction
C_2+_	Multi-carbon
FE	Faradaic efficiency
CV	Cyclic voltammetry
CV-CuO	CV-activated CuO
P-CuO	pristine CuO
HER	Hydrogen evolution reaction
XRD	X-ray diffraction
XPS	X-ray photoelectron spectroscopy
TEM	Transmission electron microscopy
SEM	Scanning electron microscopy
EIS	Electrochemical impedance spectroscopy
ECSA	Electrochemical active surface area
EBSD	Electron backscatter diffraction
GC	Gas chromatography
NMR	Nuclear magnetic resonance
LSV	Linear sweep voltammetry
SAED	Selected-area electron diffraction
RHE	Reversible hydrogen electrode
