# Supplementary material for: Hierarchical CuO Nanorods via Cyclic Voltammetry Treatment: Freestanding Electrodes for Selective CO2-to-Formate Conversion
Source: Nanomaterials (Basel). 2025 Sep 2;15(17):1349. doi: 10.3390/nano15171349 (PMC12430157; doi:10.3390/nano15171349)
Supplement: Supplementary file 1 [file nanomaterials-15-01349-s001.zip › nanomaterials-3814740-supplementary.pdf]

nanomaterials

MDPI

Supplementary Materials

Hierarchical CuO Nanorods via Cyclic Voltammetry Treatment: Freestanding Electrodes for Selective CO_2-to-Formate Conversion

Lili Wang [1,2,†], Xianlong Lu [1,2,†] and Bangwei Deng [1,2,*]

[1] Yangtze Delta Region Institute (Huzhou), University of Electronic Science and Technology of China, Huzhou 313001, China; wanglili@csj.uestc.edu.cn (L.W.); 221122270121@zjut.edu.cn (X.L.)
[2] CMA Key Open Laboratory of Transforming Climate Resources to Economy, Chongqing 401147, China
* Correspondence: bwdeng@uestc.edu.cn
† These authors contributed equally to this work.

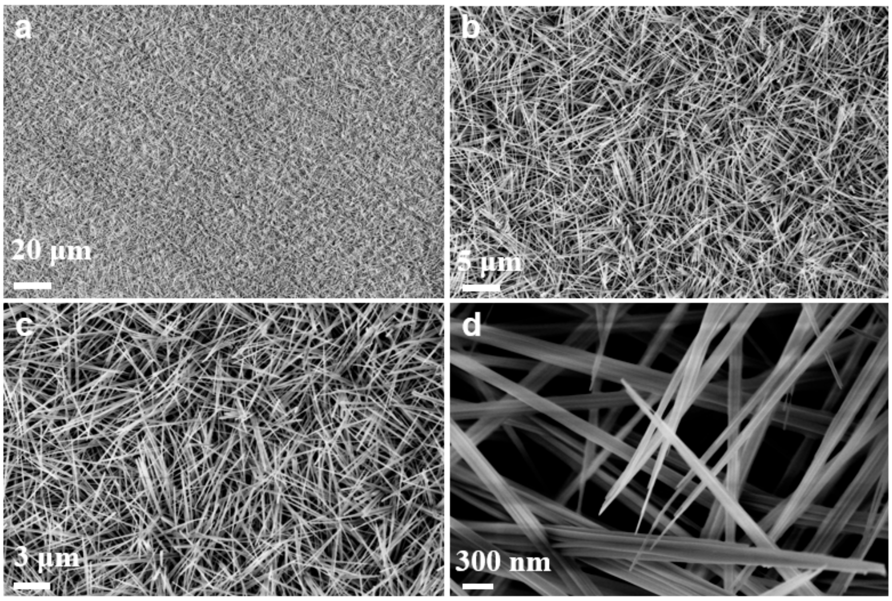

Figure S1. (a-d) SEM images of as prepared $Cu(OH)_2$ nanorod arrays at different magnifications.

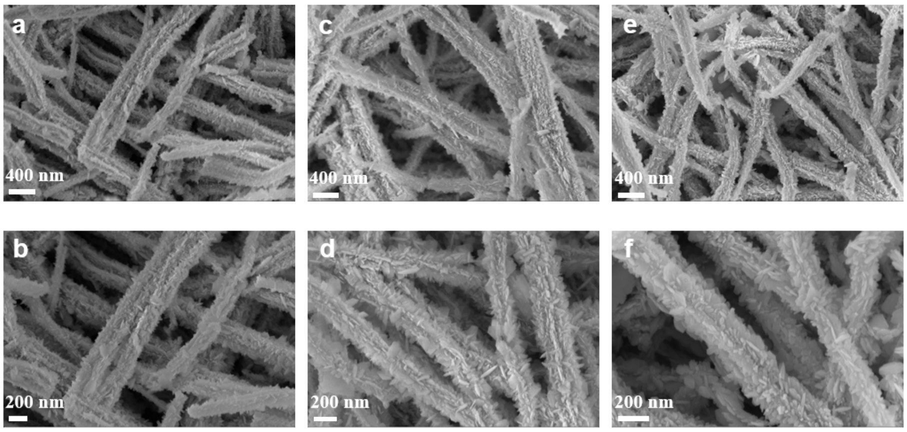

Figure S2. SEM images of as prepared CV-CuO nanorod arrays after (a-b) 2000, (c-d) 4000, and (e-f) 8000 cycles at different magnifications.

 https://doi.org/10.3390/nano15171349

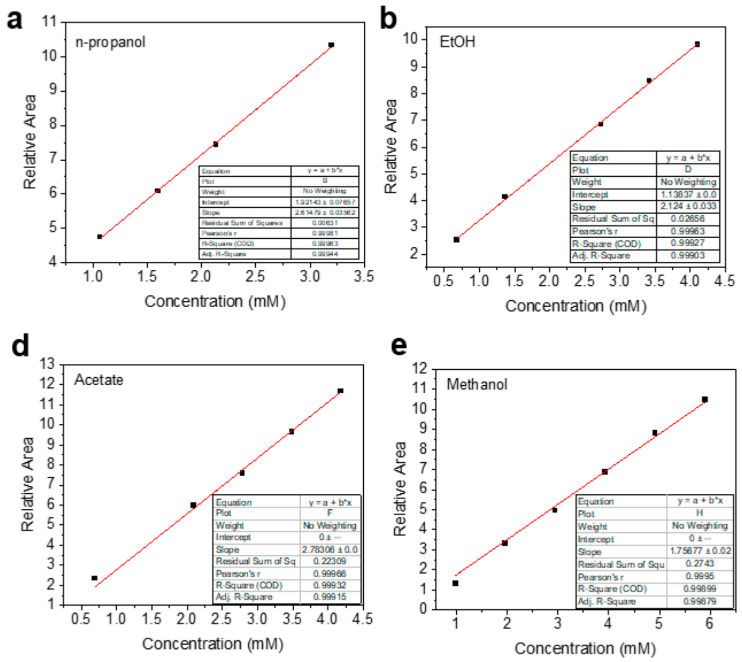

Figure S3. Calibration curves for (a) n-propanol, (b) ethanol, (c) formate, (d) acetate and (e) methanol components for 1D 1H NMR quantitative analysis.

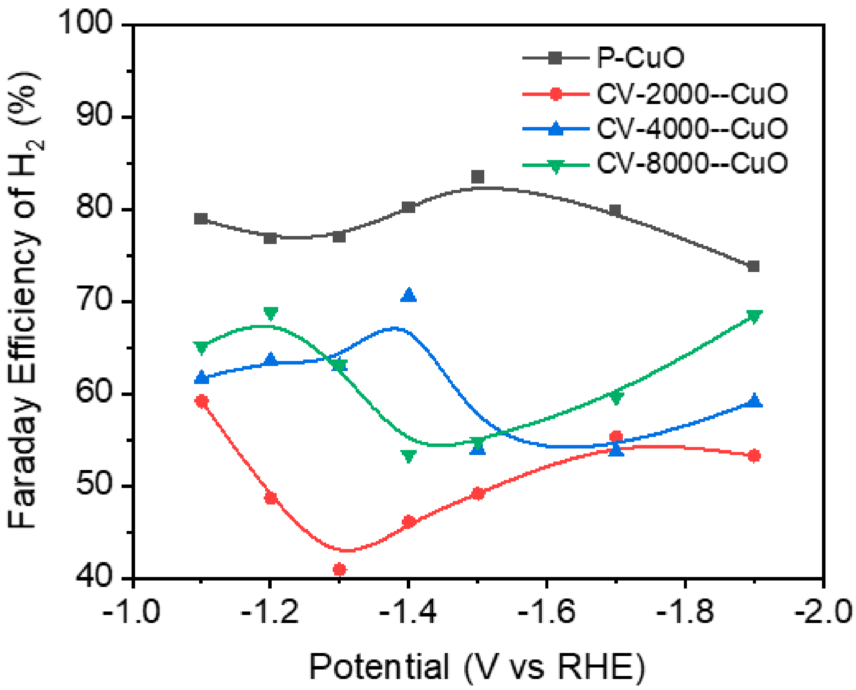

Figure S4. The corresponding potential-dependent FE curves of H_2 for P-CuO, CV-2000-CuO, CV-4000-CuO, and CV-8000-CuO nanorod arrays electrodes.

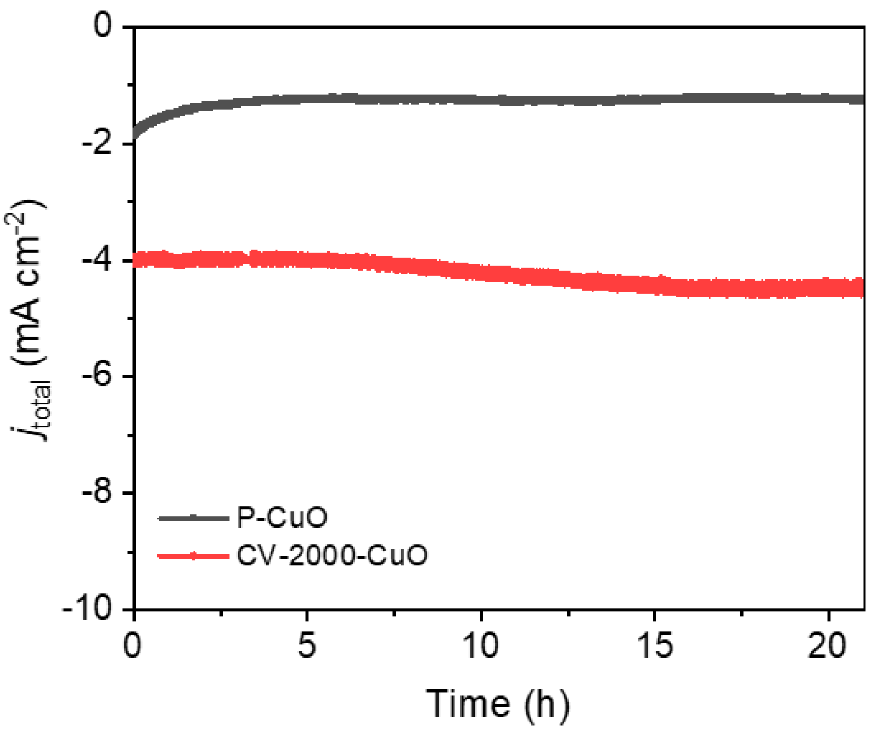

Figure S5. The stability test for P-CuO and CV-2000-CuO nanorod arrays electrodes.

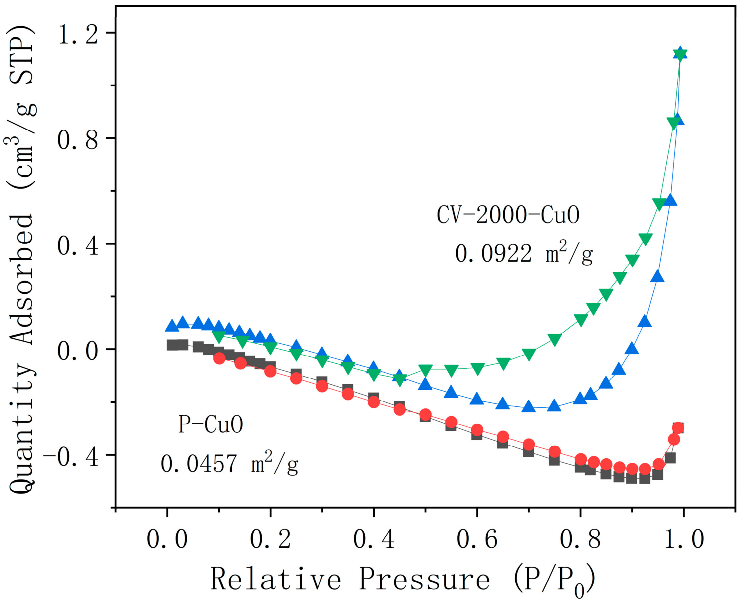

Figure S6. The BET measurements for P-CuO and CV-2000-CuO nanorod arrays electrodes.

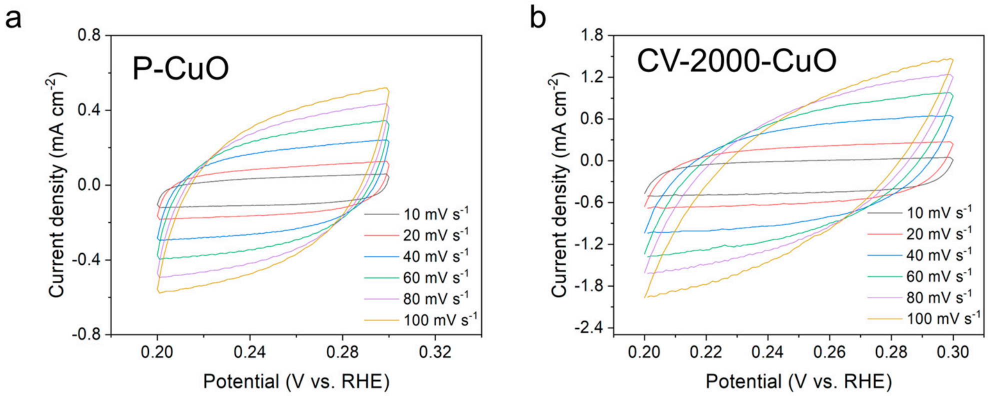

Figure S7. Double-layer capacitances. CVs taken over a range of scan rates on (a)P-CuO and (b) CV-2000-CuO respectively.

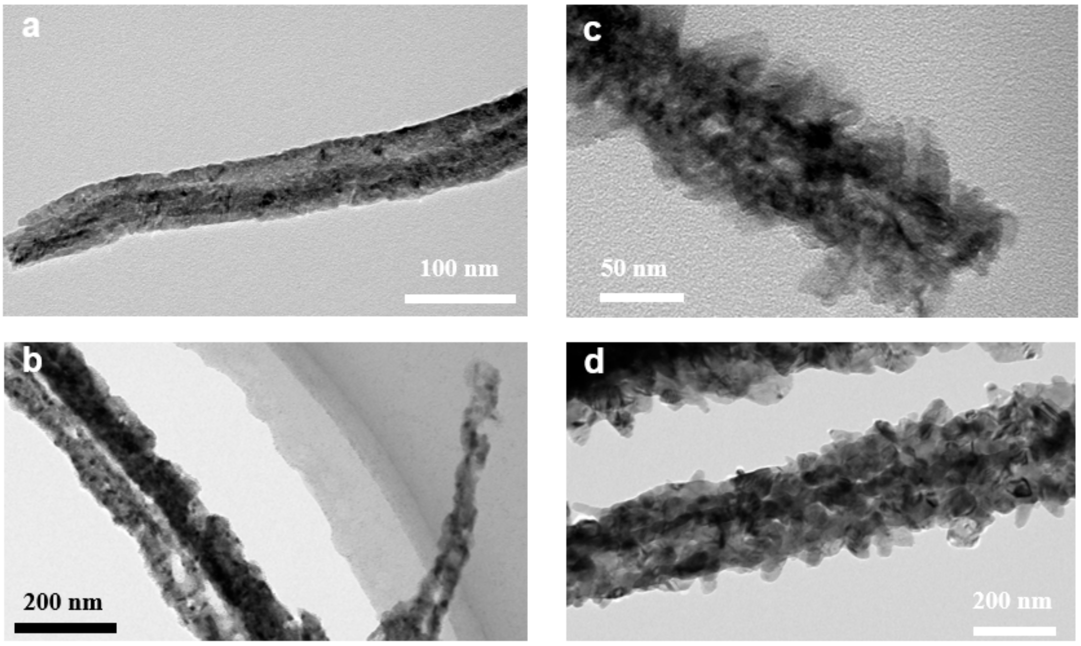

Figure S8. The high-resolution TEM images of prepared catalysts (a-b) P-CuO and (c-d) CV-2000-CuO.

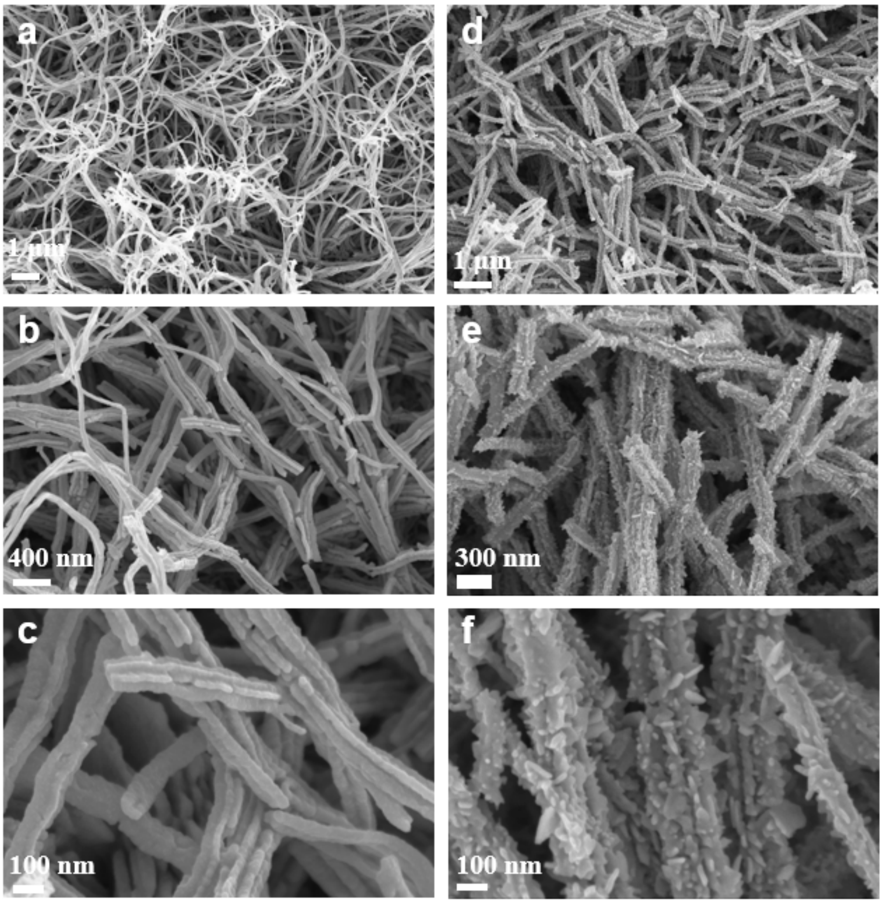

Figure S9. SEM images of (a-c) P-CuO and (d-f) CV-CuO nanorod arrays at different magnifications after electrochemical activated for 30min.

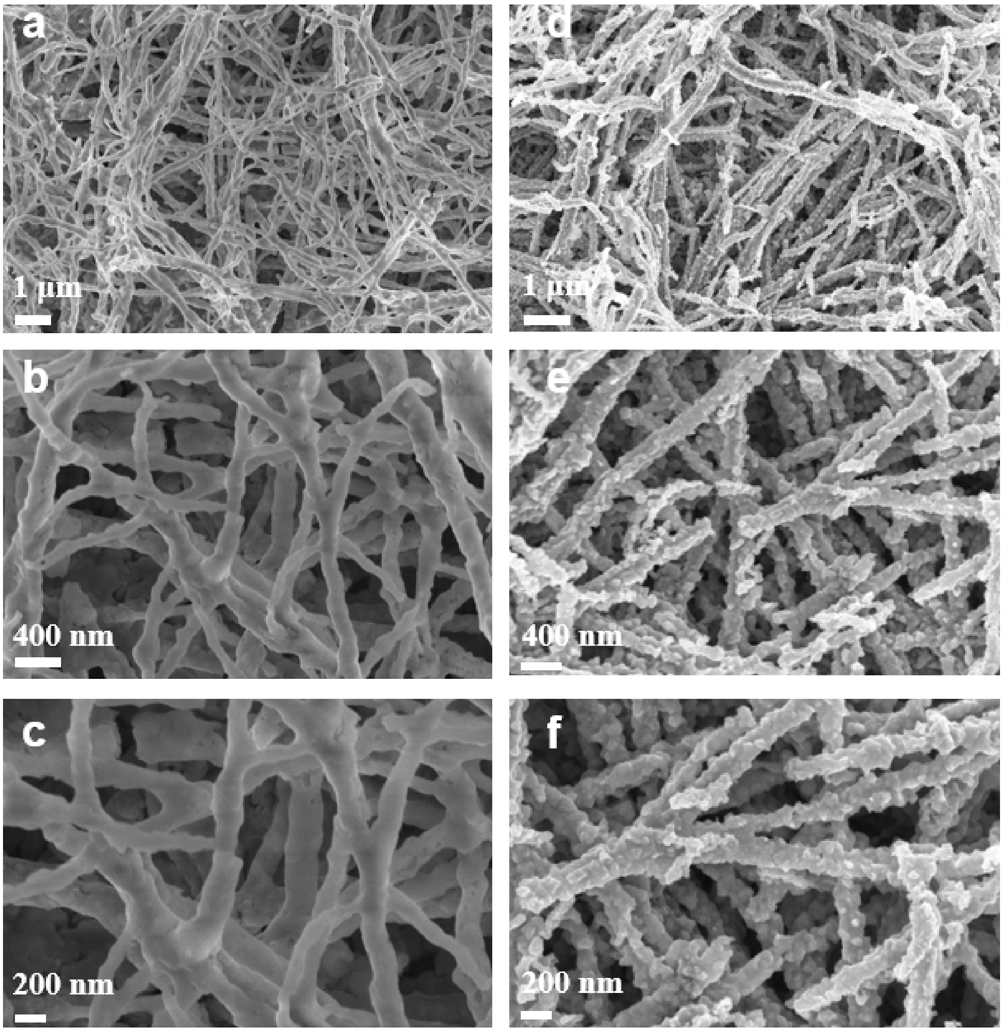

Figure S10. SEM images of (a-c) P-CuO and (d-f) CV-CuO nanorod arrays at different magnifications after CO_2RR.

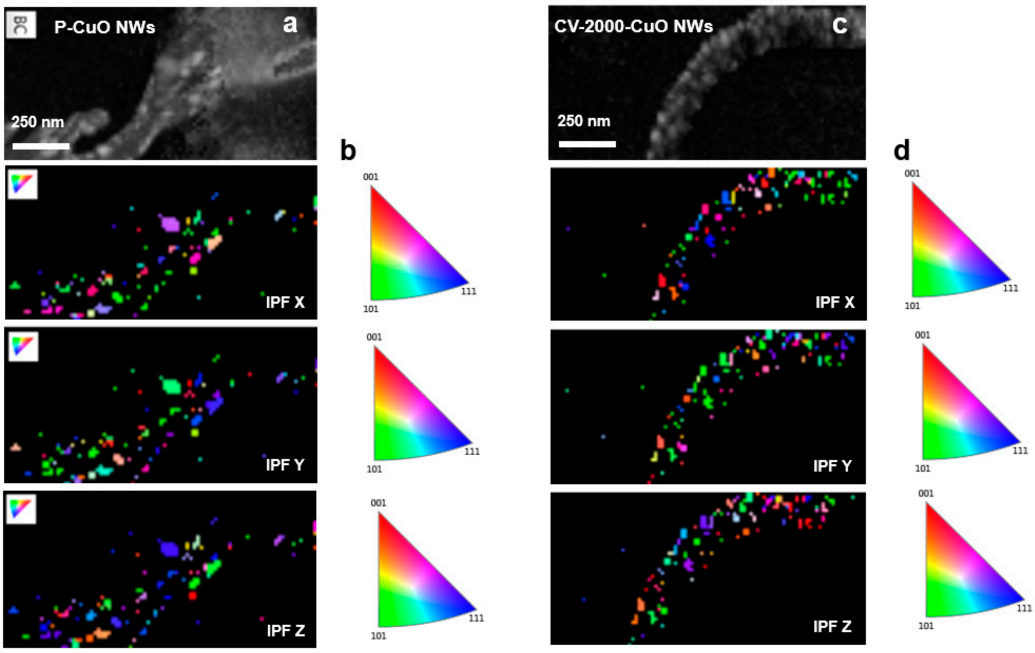

Figure S11. The SEM-SAED patterns of prepared catalysts (a-c) P-CuO and (d-f) CV-2000-CuO.

Tables

Table S1. Performances of CO_2 electrolysis to formate products over recently reported catalysts

Catalyst	Electrolyzer	Electrolyte	Potential (V vs. RHE)	Formate (FE)	Reference
CuO-derived Cu nanowire array electrodes	H-cell	0.1 M $KHCO_3$	-0.6	30%	1
Highly dense Cu nanowires.	H-cell	0.1 M $KHCO_3$	-0.6	30.7%	2
Cu Nanowire Arrays	H-cell	0.1 M $KHCO_3$	-1.1	23%	3
ECR nanowires	H-cell	0.1 M $KHCO_3$	-0.5	25%	4
Cu-HNs	H-cell	0.1 M $KHCO_3$	-0.6	38%	5
Prism-CuO microspheres	H-cell	0.5 M $KHCO_3$	-1.6	65.1%	6
CuO-derived inverse opal	H-cell	0.1 M $KHCO_3$	-0.3	47%	7
Cu-CTAB	H-cell	0.5 M $KHCO_3$	-0.5	82.3%	8
OD-Cu NAs	H-cell	0.1 M $KHCO_3$	-0.55	18%	9
Cu (111)	H-cell	0.1 M $KHCO_3$	-1.2	16%	10
Cu100-Pre	H-cell	0.1 M $KHCO_3$	-1.05	12.4%	11
CuO-SC	flow cell	1.0 M KOH	-0.37	7.8%	12
CuO-MC	flow cell	1.0 M KOH	-0.5	13.6%	12
CuO-FC	flow cell	1.0 M KOH	-0.37	9.8%	12
CV-2000-CuO	H-cell	0.1 M $KHCO_3$	-1.4	42%	This work

References

1. Ma, M.; Djanashvili, K.; Smith, W. A., Selective electrochemical reduction of CO_2 to CO on CuO-derived Cu nanowires. *Phys. Chem. Chem. Phys.* **2015,** *17*, 20861-7.

2. Raciti, D.; Livi, K. J.; Wang, C., Highly Dense Cu Nanowires for Low-Overpotential CO_2 Reduction. *Nano Lett.* **2015,** *15*, 6829-6835.

3. Ma, M.; Djanashvili, K.; Smith, W. A., Controllable Hydrocarbon Formation from the Electrochemical Reduction of CO_2 over Cu Nanowire Arrays. *Angew. Chem. Int. Ed.* **2016,** *55*, 6680-6684.

4. Cao, L.; Raciti, D.; Li, C. Y.; Livi, K. J. T.; Rottmann, P. F.; Hemker, K. J.; Mueller, T.; Wang, C., Mechanistic Insights for Low-Overpotential Electroreduction of CO_2 to CO on Copper Nanowires. *ACS Catal.* **2017,** *7*, 8578-8587.

5. Raciti, D.; Wang, Y. X.; Park, J. H.; Wang, C., Three-Dimensional Hierarchical Copper-Based Nanostructures as Advanced Electrocatalysts for CO_2 Reduction. *ACS Appl. Energy Mater.* **2018,** *1*, 2392-2398.

6. Li, D.; Huang, L. L.; Liu, T. T.; Liu, J.; Zhen, L.; Wu, J.; Feng, Y. J., Electrochemical reduction of carbon dioxide to formate via nano-prism assembled CuO microspheres. *Chemosphere* **2019,** *237*, 124527.

7. Nguyen-Phan, T. D.; Wang, C. J.; Marin, C. M.; Zhou, Y. Y.; Stavitski, E.; Popczun, E. J.; Yu, Y.; Xu, W. Q.; Howard, B. H.; Stuckman, M. Y.; Waluyo, I.; Ohodnicki, P. R.; Kauffman, D. R., Understanding three-dimensionally interconnected porous oxide-derived copper electrocatalyst for selective carbon dioxide reduction. *J. Mater. Chem. A* **2019,** *7*, 27576-27584.

8. Tao, Z. X.; Wu, Z. S.; Wu, Y. S.; Wang, H. L., Activating Copper for Electrocatalytic CO_2 Reduction to Formate via Molecular Interactions. *ACS Catal.* **2020,** *10*, 9271-9275.

9. Wang, Y. X.; Niu, C. L.; Zhu, Y. C.; He, D.; Huang, W. X., Tunable Syngas Formation from Electrochemical CO_2 Reduction on Copper Nanowire Arrays. *ACS Appl. Energy Mater.* **2020,** *3*, 9841-9847.

10. Kim, H.; Lee, J.; Lee, S.; Park, S.; Lee, Y.; Lee, G.; Jeon, H.S.; Han, M.H.; Jin, S.; Lee, H.W.; et al. Selective Electrosynthesis of Methanol from CO_2 Over $Cu/Cu_2P_2O_7$ Via the Formate Pathway. Adv. Mater. **2025**, 2501021.

11. Jang, J.; Delmo, E.P.; Chen, W.; Sun, Z.; Wan, D.H.C.; Liu, Y.; Zhu, S.; Wang, Y.; Li, T.; Huang, H.; et al. Metal‐Organic Framework‐Derived Partially Oxidized Cu Electrocatalysts for Efficient CO_2 Reduction Reaction Toward C_{2+} Products. Carbon Energy **2025**, e70019.

12. Yang, C.; Shen, H. C.; Guan, A. X.; Liu, J. L.; Li, T. F.; Ji, Y. L.; Al-Enizi, A. M.; Zhang, L. J.; Qian, L. P.; Zheng, G. F., Fast cooling induced grain-boundary-rich copper oxide for electrocatalytic carbon dioxide reduction to ethanol. *J. Colloid Interface Sci.* **2020**, 570, 375-381.

Disclaimer/Publisher's Note: The statements, opinions and data contained in all publications are solely those of the individual author(s) and contributor(s) and not of MDPI and/or the editor(s). MDPI and/or the editor(s) disclaim responsibility for any injury to people or property resulting from any ideas, methods, instructions or products referred to in the content.
